# *Cox-LASSO* Analysis for Hospital Mortality in Patients With Sepsis Received Continuous Renal Replacement Therapy: A MIMIC-III Database Study

**DOI:** 10.3389/fmed.2021.778536

**Published:** 2022-02-10

**Authors:** Chunxia Wang, Jianli Zheng, Jinxia Wang, Lin Zou, Yucai Zhang

**Affiliations:** ^1^Department of Critical Care Medicine, Shanghai Children's Hospital, Shanghai Jiao Tong University, Shanghai, China; ^2^Institute of Pediatric Critical Care, Shanghai Jiao Tong University, Shanghai, China; ^3^Clinical Research Unit, Shanghai Children's Hospital, Shanghai Jiao Tong University, Shanghai, China; ^4^Institute of Pediatric Infection, Immunity, and Critical Care Medicine, Shanghai Jiao Tong University School of Medicine, Shanghai, China; ^5^Institute of Medical Information Engineering, University of Shanghai for Science and Technology, Shanghai, China

**Keywords:** MIMIC-III, Sepsis-3.0, *LASSO*, *Cox* regression, mortality, CRRT

## Abstract

**Background:**

Sepsis remains the leading cause of mortality in-hospital in the intensive care unit (ICU). Continuous renal replacement therapy (CRRT) is recommended as an adjuvant therapy for hemodynamics management in patients with sepsis. The aim of this study was to develop an adaptive least absolute shrinkage and selection operator (*LASSO*) for the *Cox* regression model to predict the hospital mortality in patients with Sepsis-3.0 undergoing CRRT using Medical Information Martin Intensive Care (MIMIC)-III v1.4.

**Methods:**

Patients who met the Sepsis-3.0 definition were identified using the MIMIC-III v1.4. Among them, patients who received CRRT during ICU hospitalization were included in this study. According to the survival status, patients were split into death or survival group. Adaptive *LASSO* for the *Cox* regression model was constructed by STATA software. At last, nomogram and Kaplan-Meier curves were drawn to validate the model.

**Results:**

A total of 181 patients who met Sepsis 3.0 criteria received CRRT were included in the study, in which, there were 31 deaths and 150 survivals during hospitalization, respectively. The overall in-hospital mortality was 17.1%. According to the results of multivariate *Cox-LASSO* regression analysis, use of vasopressor, international normalized ratio (INR) ≥1.5, and quick sequential organ failure assessment (qSOFA) score were associated with hospital mortality in patients with sepsis who underwent CRRT, but lactate level, mechanical ventilation (MV) support, PaO_2_/FiO_2_, platelet count, and indicators of acute kidney injury (AKI), such as blood urea nitrogen (BUN) and creatinine, were not independently associated with hospital mortality after adjusted by qSOFA. The risk nomogram and Kaplan-Meier curves verified that the use of vasopressor and INR ≥1.5 possess significant predictive value.

**Conclusions:**

Using the *Cox-LASSO* regression model, use of vasopressor, INR ≥1.5, and qSOFA score are found to be associated with hospital mortality in patients with Sepsis-3.0 who received CRRT. This finding may assist clinicians in tailoring precise management and therapy for these patients who underwent CRRT.

## Introduction

Sepsis is a major condition with high morbidity and mortality in intensive care unit (ICU) patients (1). Severe sepsis and septic shock are characterized by vasoplegia and alterations of microcirculation, resulting in aggressively hemodynamic alterations that render the patient hypotensive or with organ dysfunction (2–5). During sepsis, fluid responsiveness or the use of vasopressors could guide fluid administration (6), but the response to therapy is highly variable (7, 8). Improvement of hemodynamic may not be related to the improvement of microcirculation (4, 9). Septic shock is defined as a microcirculation disease, and many trials showed that the severity of microvascular alterations is associated with outcomes in patients with septic shock (10–14). Evaluation of the response for hemodynamic management is critical for the prognosis of sepsis.

According to the 2020 Surviving Sepsis Campaign (SSC) guidelines, renal replacement therapy (RRT)/continuous RRT (CRRT) has emerged as the preferred modality for critically ill patients to treat acute kidney injury (AKI), fluid overload, particularly, those with hemodynamic instability who are unresponsive to fluid restriction and diuretic therapy (15). In adult septic patients who underwent RRT, microcirculation was improved despite no significant variation in macro-hemodynamics (16). Sepsis-induced aggressively hemodynamic alterations are mainly caused by endothelial dysfunction resulting in the activation of inflammation and coagulation processes (5, 17). CRRT plays an important role in removing toxins and inflammatory factors, and higher TNF-α removal could be related to the lower mortality observed in patients with AKI (18). In addition, patients with sepsis suffer from a higher risk of bleeding and clotting. Anticoagulation is necessary for the effective delivery of CRRT, and anticoagulation for CRRT should be adapted to the patient's characteristics (19). Given the complex roles of CRRT in improving inflammatory response, fluid management, and anticoagulation involved in CRRT management, assessment of the prognosis in patients with sepsis who underwent CRRT could be especial. Until now, the risk factors of worse prognosis in patients with sepsis who received CRRT are limited to be reported.

In the current study, we conducted a retrospective study based on Medical Information Martin Intensive Care (MIMIC) III v1.4 to develop a model based on the potential risk factors related to the outcome of patients with sepsis who need CRRT. The results could be helpful for clinicians to make precise management of these patients.

## Methods

### Database and Study Population

Study data were acquired from the MIMIC-III database v1.4, which encompasses > 60,000 ICU admissions between 2001 and 2012 for > 46,000 unique patients at Beth Israel Deaconess Medical Center (BIDMC) in Boston, Massachusetts between 2001 and 2012 (20). The information available in MIMIC-III includes dates of admission to the ICU and hospital, demographic, clinical features, laboratory and microbiology test results, fluid balance, critical illness scores, diagnosis codes, and hospital mortality. Use of the MIMIC-III database was approved by the Institutional Review Boards of BIDMC and the Massachusetts Institute of Technology.

**Figure 1:**
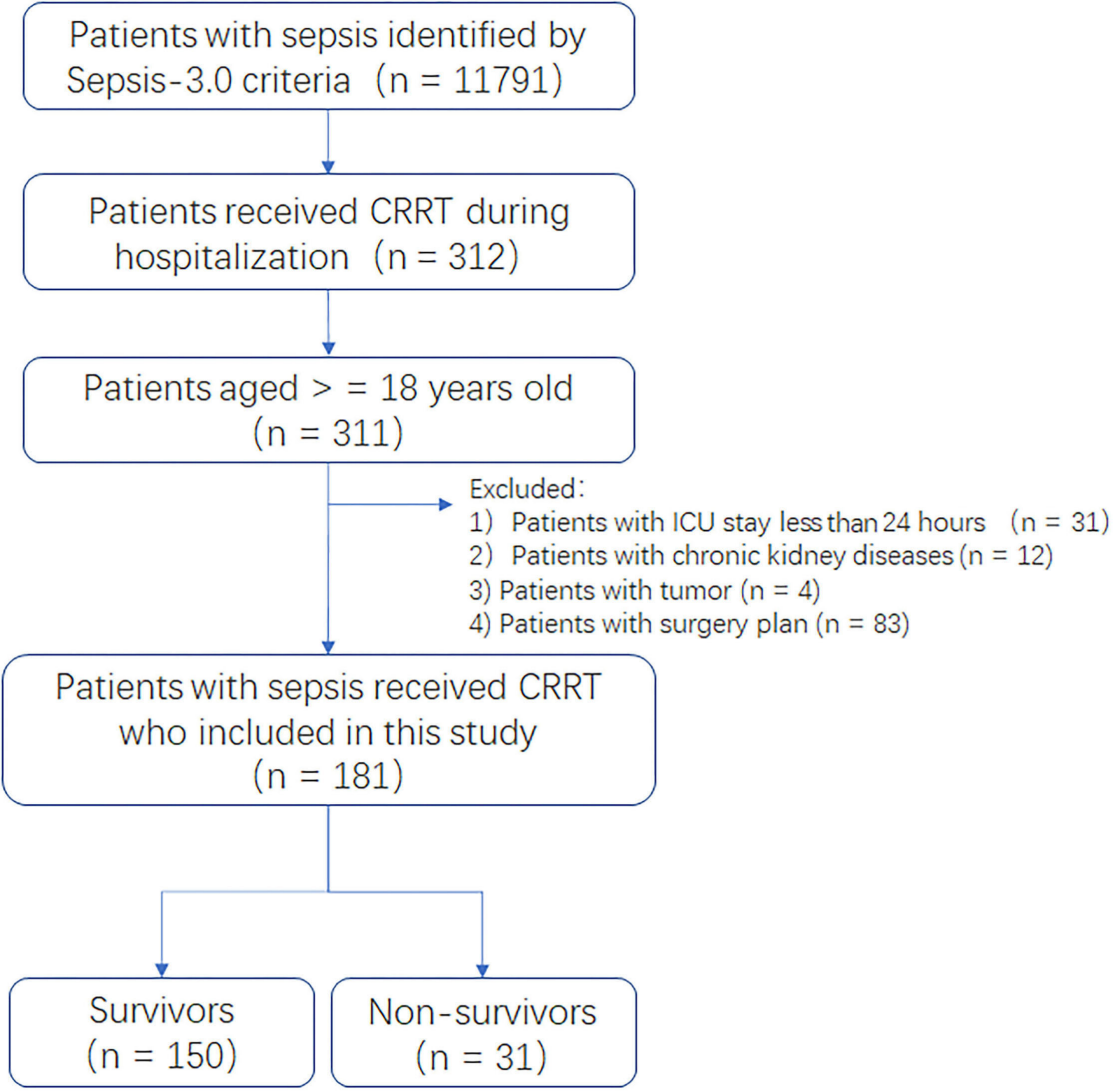
Flow chart of patient selection and data extraction.

Firstly, data extraction adhered to the original Sepsis-3.0 definition as closely as possible (21, 22). According to the report of Johnson (23), the patients who fulfilled the Sepsis-3.0 criteria were automatically extracted using pgAdmin PostgreSQL tools (version 1.22.1). Of these patients, patients who aged over 18-year-old received CRRT during hospitalization were included. We excluded those with conditions who may be associated with hospital mortality, such as: (1) the length of ICU stay <24 h; (2) with chronic kidney disease (International Classification of Diseases [ICD]9-code: 5859); (3) metastatic cancer and solid tumor without metastasis (metastatic cancer: icd9_code: 1960–1991, 20970–20975, 20979, 78951; solid tumor without metastasis: icd9_code: 1400–1729, 1740–1759, 179–1958, 20900–20924, 20925–2093, 20930–20936, 25801–25803); or (4) surgery plan. Patients were divided into two groups based on the record of the hospital expire flag (in-hospital death recorded in the hospital database). The detailed process of patients' selection and data extraction is shown in Figure 1.

### Outcomes

The primary outcome was hospital mortality at the first ICU admission. The secondary outcomes were the length of ICU and hospital stay, use of vasopressor, and mechanical ventilation (MV) support.

### Data Extraction and Variables Collection

Variables extracted from MIMIC-III database v1.4 included demographics, severity accessed by SOFA, qSOFA, systemic inflammatory response syndrome (SIRS), and Logistic Organ Dysfunction System (LODS) scores, source of patients, vital signs, such as heart rate (HR), systolic blood pressure (BP), diastolic BP, mean arterial pressure (MAP), temperature, respiratory rate (RR), arterial blood gas, such as oxyhemoglobin saturation (SpO_2_) and PaO_2_/FiO_2_, serum laboratory variables that include the minimum of albumin, platelet, the maximum of bilirubin, creatinine, lactate, international normalized ratio (INR), blood urea nitrogen (BUN), and white blood cells (WBC), and the test results of blood infection. Furthermore, oxygen therapy support mode, duration of ventilation, use of vasopressor, and vasopressor duration were accessed. Patient demographics and all necessary variables were calculated using data from the first 24 h of the ICU stay. Furthermore, we set categorical variables based on the values of laboratory indexes within 24 h after ICU admission as below: (1) systolic BP <100 mmHg, (2) whether or not need vasopressor, (3) INR ≥1.5, (4) platelet <100 × 10^9^/L, (5) lactate ≥4 μmol/L, (6) impaired pulmonary function was defined as PaO_2_/FiO_2_ >300 mmHg, 200 mmHg < PaO_2_/FiO_2_ ≤ 300 mmHg, 100 mmHg < PaO_2_/FiO_2_ ≤ 200 mmHg, and PaO_2_/FiO_2_ ≤ 100 mmHg. Ultimately, we obtained the list of data based on anonymized patients with Sepsis-3.0 who received CRRT.

### Statistical Analysis

All the data analyses were conducted using STATA 15.0 MP (College Station, TX, USA). Variables were displayed and compared between survivors and non-survivors. Normally and non-normally distributed continuous variables were expressed as the mean ± SD and the median (interquartile range, IQR), respectively. Continuous variables of normal distribution were tested by Student's *t-*test. Mann-Whitney *U*-test was used to compare continuous data of non-normally distribution. Categorical variables were summarized as numbers or percentage and assessed using the Chi-square test. A *p* < 0.05 was defined as statistically significant.

Cox survival analysis and least absolute shrinkage and selection operator (LASSO) regression univariable regression analyses were performed to assess the association of each variable with hospital mortality, and values of *p* < 0.05 were selected as a candidate variable. The method of LASSO was used to select predictors. Multivariate *Cox* regression was further performed to assess the prognostic value of selected variables, with qSOFA as an adjustment factor. The hazard ratio (HR) and 95% CI were estimated by *Cox* proportional hazards regression model.

### Construction and Validation of a Prognostic Nomogram for Hospital Mortality

Nomogram were constructed to calculate an individual's probability of hospital mortality by using STATA software. In the nomogram, the patient was scored according to the variables entered multivariate *Cox* proportional hazards regression model. The final sum of the scores was expected to be the corresponding hospital mortality probability. Kaplan-Meier curves were drawn and compared the differences in hospital mortality between groups divided by the variables of the nomogram.

## Results

### Baseline Characteristics

There were 11,791 patients with Sepsis-3.0 between 2008 and 2012. In this cohort, 312 patients received CRRT during hospitalization. One patient aged less 18-year-old, 31 cases with the length of ICU stay <24 h, 12 patients with chronic kidney disease, 4 patients with tumor, and 83 patients with surgery plan were excluded. Finally, there were 181 patients with Sepsis-3.0 who underwent CRRT during hospitalization, in which, there were 31 deaths and 150 survivals during hospitalization, respectively.

In these patients, the age, gender, ethnicity, first service type, blood infection, and the length of ICU stay showed no significant difference between survival and non-survival groups. The ratio of MV was higher in non-survivors than survivors, and the length of hospital stay was shorter in non-survivors than survivors (Table 1).

**Table 1:** Baseline characteristics in patients with sepsis who received CRRT.

**Parameters**	**Total (*n* = 181)**	**Survivors (*n* = 150)**		**Non-survivors (*n* = 31)**	***P***
**Demographic variables**					
Gender male, *n* (%)	109 (60.2)	89 (59.3)		20 (64.5)	0.591
Age, year, mean (SD)	61.9 (14.82)	61.2 (15.11)		65.0 (13.12)	0.196
Ethnicity, n 0.530					
White	101	81		20	0.283
Black	34	32		2	0.053
Hispanic	12	11		1	0.403
Others	34	26		8	0.272
**Severity, median (IQR)**					
SOFA	7 (5–10)	6 (5–9)		11 (8–16)	<0.001
qSOFA	2 (1–2)	2 (1–2)		2 (2–3)	<0.001
SIRS	3 (2–4)	3 (2–3)		3 (2–4)	0.033
LODS	6 (5–8)	6 (4–7)		9 (7–13)	<0.001
First service, *n*					0.546
CMED	24	18		6	
MED	152	127		25	
NMED	2	2		0	
OMED	3	3		0	
Blood infection, *n*	68	54		14	0.338
Mechanical ventilation, *n* (%)	76 (42)	53 (35.3)		23 (74.2)	<0.001
Length of ICU stay, days, median (IQR)	2.9 (1.8–5.7)	2.8 (1.8–5.7)		3.3 (1.7–7.2)	0.778
Length of hospital stay, days, median (IQR)	7.7 (4.1–4.0)	9.8 (5.1–15.6)		3.4 (1.6–9.8)	<0.001

*SOFA, sequential organ failure assessment; qSOFA, Quick SOFA; SIRS, systemic inflammatory response syndrome; LODS, Logistic Organ Dysfunction System*.

### Laboratory Indexes Within the First 24 h After ICU Admission

Minimum systolic BP, minimum diastolic BP, minimum MAP, maximum RR, maximum lactate, maximum creatinine, maximum bilirubin, minimum platelet, maximum INR, minimum albumin, ratio of vasopressor needed, and respiratory support were significantly different between survivors and non-survivors. However, maximum HR, maximum temperature, maximum glucose, maximum BUN, minimum WBC, maximum WBC, SpO_2_, PaO_2_/FiO_2_, vasopressor duration, and ventilation durations showed no significant difference between the two groups (Table 2).

**Table 2:** Laboratory indexes within 24 h after ICU admission in patients with sepsis who received CRRT.

**Parameters**	**Total (*n* = 181)**	**Survivors (*n* = 150)**	**Non-survivors (*n* = 31)**	***P***
Vital signs, median (IQR) if not otherwise specified				
Maximum heart rate (/min), mean (SD)	105 (22)	104 (22)	109 (21)	0.241
Minimum systolic BP (mmHg)	87 (77–103.5)	90 (81–107)	73 (62–80)	<0.001
Systolic BP group, (mmHg)				0.002
Systolic BP ≥ 100, *n*	53	51	2	
Systolic BP <100, *n*	128	99	29	
Minimum diastolic BP (mmHg)	40 (33–49)	41 (35–49)	35 (27–44)	0.005
Diastolic BP group, (mmHg)				0.370
Diastolic BP ≥ 60, *n*	20	18	2	
Diastolic BP <60, *n*	161	132	29	
Minimum MAP (mmHg)	54 (47–63)	55.5 (48–64)	47 (40–51)	<0.001
MAP group, (mmHg)				0.327
MAP ≥ 70, *n*	28	25	3	
MAP <70, *n*	153	125	28	
Maximum respiratory rate (/min)	28 (23–32)	27 (23–31)	33 (28–35)	<0.001
Respiratory rate group, (/min)				0.226
Respiratory rate ≤ 20, *n*	16	15	1	
Respiratory rate > 20, *n*	165	135	30	
Maximum temperature (°C), mean (SD)	37.4 (1.0)	37.5 (0.9)	37.3 (1.4)	0.432
Serum laboratory variables, median (IQR) if not otherwise specified				
Maximum lactate (μmol/L)	2.2 (1.4–4.6)	1.9 (1.4–3.3)	4.8 (2–9.9)	<0.001
Lactate group, (μmol/L)				0.068
Lactate <4, *n*	97	85	12	
Lactate ≥ 4, *n*	84	65	19	
Maximum creatinine (μmol/L)	5.4 (3.7–8.2)	5.8 (3.6–9)	4.7 (3.8–5.7)	0.036
Maximum glucose (mg/dL)	166 (122–243)	161 (121–230)	213 (132–290)	0.062
Maximum bilirubin (mg/dL)	0.65 (0.4–1.5)	0.5 (0.3–0.9)	1.8 (0.6–4.1)	0.001
Bilirubin group, (mg/dL)				0.797
Bilirubin <4, *n*	119	98	21	
Bilirubin ≥ 4, *n*	62	52	10	
Minimum platelet (×10^9^/L)	167 (106–229)	174.5 (111–30.5)	121 (76–182)	0.045
Platelet group, (×10^9^/L)				0.038
Platelet ≥ 100, *n*	142	122	20	
Platelet <100, *n*	39	28	11	
Maximum INR	1.4 (1.2–1.9)	1.3 (1.2–1.6)	2 (1.4–2.7)	<0.001
INR group				0.004
INR <1.5, *n*	95	86	9	
INR ≥ 1.5, *n*	86	64	22	
Maximum BUN, (mmol/L)	52.5 (41–79)	53 (41–79)	49 (43–81)	0.887
Minimum WBC (×10^9^/L)	8.6 (5.8–13.7)	8.3 (5.9–13.2)	9.1 (5.4–14.6)	0.894
Maximum WBC (×10^9^/L)	11.8 (7.8–19.5)	11.4 (7.8–17.9)	14.8 (7.5–20.4)	0.327
Minimum albumin (g/dL)	3.1 (2.6–3.8)	3.25 (2.7–3.8)	2.6 (2.3–3.1)	0.001
Albumin group, (g/dL)				0.252
Albumin ≥ 4, *n*	87	75	12	
Albumin <4, *n*	94	75	19	
Pulmonary parameters, median (IQR) if not otherwise specified				
SpO_2_	96 (92.5–97.5)	96 (93–98)	95.5 (89.5–97)	0.184
PaO_2_/FiO_2_, mmHg	112.5 (74.5–91)	122(80–196.7)	90 (63–140)	0.132
Impaired pulmonary function group				<0.001
PaO_2_/FiO_2_ ≥ 300, *n*	119	108	11	
300 < PaO_2_/FiO2 ≤ 200, *n*	8	5	5	
200 < PaO_2_/FiO_2_ ≤ 100, *n*	25	20	5	
PaO_2_/FiO_2_ <100, *n*	29	17	12	
Vasopressor				<0.001
No, *n* (%)	109 (60.2)	104 (69.3)	5 (16.1)	
Yes, *n* (%)	72 (39.8)	46 (30.7)	26 (83.9)	
Vasopressor duration, hours	44.8 (23.0–20.0)	41.8(15.5–24.2)	63.8 (27.6–15.7)	0.281
Respiratory support model, *n* (%)				<0.001
None, *n* (%)	19 (10.5)	19 (12.7)	0 (0)	
Oxygen therapy, *n* (%)	82 (45.3)	76 (50.7)	6 (19.4)	
Mechanical ventilation, *n* (%)	80 (44.2)	55 (36.7)	25 (80.6)	
Ventilation durations, hours	65.6 (27.9–67.5)	82 (30.8–89.8)	60 (27.4–98.2)	0.338

*SD, standard deviation; IQR, inter quartile range; BP, blood pressure; SpO_2_, pulse oxygen saturation; PaO_2_/FiO_2_, the ratio of the partial pressure of oxygen in arterial blood (PaO_2_) to the inspired oxygen fraction (FiO_2_); INR, international normalized ratio*.

### Relationship Between Clinical and Laboratory Indexes and Hospital Mortality

Overall hospital mortality was 17.1% (31/181). The ratio of MV needed was 44.2% (80/181), and the ratio of use of vasopressor was 39.8% (72/181). The hospital mortality was 36.1% (26/72) in patients who received vasopressor and 4.6% (5/109) in patients without vasopressor support (*p* < 0.001). In a subgroup of patients who received MV support, the hospital mortality was 31.3 (25/80), which was significantly higher than that 7.3% (6/82) in patients with oxygen therapy, and all 19 patients without any oxygen therapy were survival.

### Identification of Risk Factors of Hospital Mortality by *Cox-LASSO* Analysis

**Figure 2:**
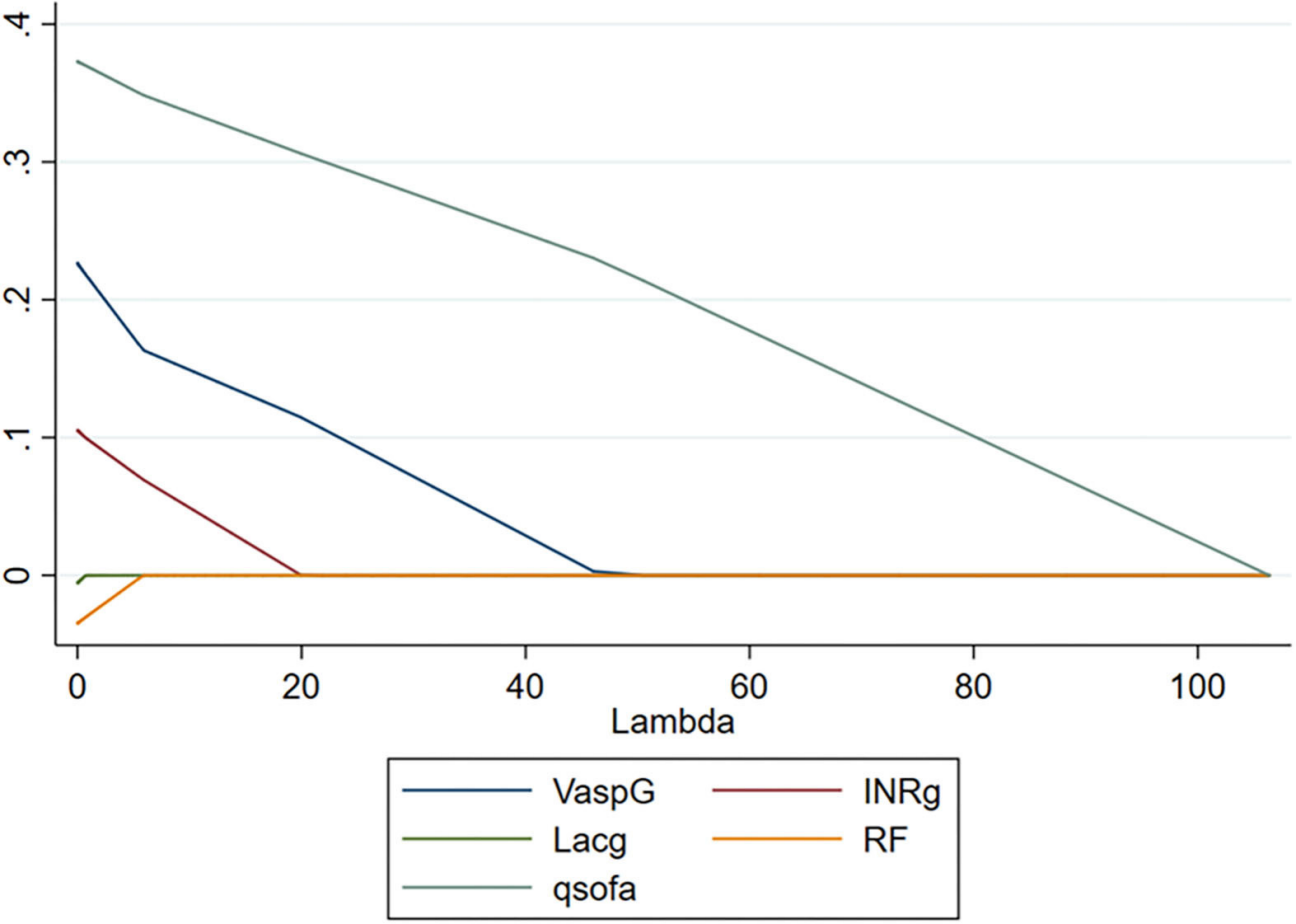
LASSO regression analysis for hospital mortality in patients with Sepsis-3.0 who received CRRT. VaspG (blue line): use of vasopressor; INRg (purple line): INR >1.5; Lacg (green line): lactate ≥4 μmol/L; RF (orange line): severity of impaired pulmonary function (defined as PaO_2_/FiO_2_ (PF) >300 mmHg, 200 mmHg < PF ≤ 300 mmHg, 100 mmHg < PF ≤ 200 mmHg, PF ≤ 100 mmHg); qSOFA (dark green line): qSOFA score. LASSO, least absolute shrinkage and selection operator; CRRT, continuous renal replacement therapy; qSOFA, quick sequential organ failure assessment; INR, international normalized ratio.

According to the results of Table 2, laboratory variables and categorical variables with statistically significant differences between survivors and non-survivors were entered in the Univariate *Cox* analysis. The results showed that systolic BP <100 mmHg, the use of vasopressor, INR ≥1.5, maximum lactate, maximum creatinine, and impaired severity of pulmonary function were associated with hospital mortality in patients with sepsis undergoing CRRT (all *p* < 0.05; Table 3). Furthermore, *LASSO* regression analysis was used to screen these variables. Adaptive *LASSO* regression analysis indicated that the categorical variables, such as the use of vasopressor, INR ≥ 1.5, impaired severity of pulmonary function, but not the absolute values of laboratory indexes, were entered multivariate *Cox* regression model (Figure 2). Finally, multivariate *Cox* regression model based on the adaptive LASSO displayed that the use of vasopressor and INR ≥ 1.5 were risk factors of hospital mortality in patients with Sepsis-3.0 who received CRRT adjusted by qSOFA (Table 4).

**Table 3:** Univariate *Cox* analysis of factor related to hospital mortality in patients with sepsis received CRRT.

**Parameters**	***HR* (95% *CI*)**	***P***
Minimum systolic BP	0.956 (0.938–0.976)	<0.001
Systolic BP <100 mmHg	5.327 (1.268–22.388)	0.022
Use of vasopressor	6,860 (2.622–17.947)	<0.001
Maximum INR	1.231 (1.127–1.346)	<0.001
INR ≥ 1.5	2.639 (1.208–5.767)	0.015
Minimum platelet	0.998 (0.995–1.001)	0.270
Platelet <100 *10^9^/L	1.890 (0.901–3.967)	0.092
Maximum lactate	1.140 (1.071–1.213)	<0.001
Lactate ≥ 4 μmol/L	2.017 (0.978–4.158)	0.057
Maximum creatinine	0.873 (0.772–0.987)	0.031
PaO_2_/FiO_2_	0.997 (0.991–1.002)	0.244
Severity of impaired pulmonary function	1.580 (1.203–2.076)	0.001
qSOFA	2.523 (1.455–4.374)	0.001

*HR, hazard ratio; CI, confidence interval; BP, blood pressure; PaO_2_/FiO_2_, the ratio of the partial pressure of oxygen in arterial blood (PaO_2_) to the inspired oxygen fraction (FiO_2_); INR, International normalized ratio; qSOFA, quick sequential organ failure assessment*.

**Table 4:** Multivariate *Cox* analysis of factor related to hospital mortality based on LASSO regression in patients with sepsis received CRRT.

**Parameters**	***HR* (95% *CI*)**	***P***
Use of vasopressor	4.564 (1.575–13.223)	0.005
INR ≥ 1.5	2.475 (1.114–5.497)	0.026
Severity of impaired pulmonary function	1.066 (0.782–1.454)	0.685
qSOFA	2.514 (1.322–4.780)	0.005

*HR, hazard ratio; CI, confidence interval; INR, International normalized ratio; qSOFA, quick sequential organ failure assessment; Continuous renal replacement therapy*.

### A New Prognostic Nomogram for Patients With Sepsis-3.0 Who Underwent CRRT

**Figure 3:**
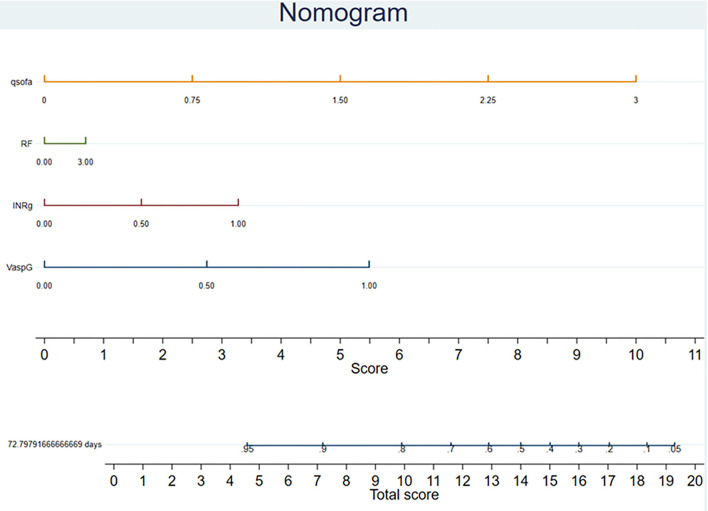
Top features were selected using multivariate *Cox-LASSO* regression analysis and the corresponding variable importance score. The X-axis indicates the importance score, which is the relative number of a variable that is used to distribute the data, the Y-axis indicates the top-weighted variables. qSOFA (orange line): quick sequential organ failure assessment; RF (green line): impaired severity of pulmonary function (defined as PaO_2_/FiO_2_ [PF]) > 300 mmHg, 200 mmHg < PF ≤ 300 mmHg, 100 mmHg < PF ≤ 200 mmHg, PF ≤ 100 mmHg). INRg (purple line): INR > 1.5; VaspG (blue line): use of vasopressor. LASSO, least absolute shrinkage and selection operator; CRRT, continuous renal replacement therapy; qSOFA, quick sequential organ failure assessment; INR, international normalized ratio.

To provide a quantitative method for clinical outcome prediction, we constructed a prognostic nomogram, such as the use of vasopressor, INR ≥ 1.5, the severity of impaired pulmonary function, and qSOFA, to predict the hospital mortality of patients with Sepsis-3.0. As shown in Figure 3, total scores were derived from the sum of the individual scores of various risk factors. In this nomogram, a higher total number of points indicated worse hospital mortality.

### Stratified Analysis of Prognostic Factors Using Kaplan-Meier Curves

**Figure 4:**
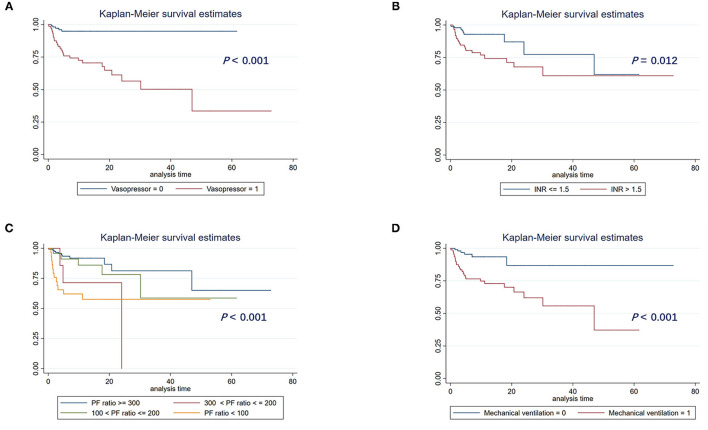
Kaplan-Meier survival curve analysis of hospital mortality in patients stratified by prognostic factors based on *Cox-LASSO* analysis. **(A)** With vs. without vasopressor; **(B)** INF ≤ 1.5 vs. INR >1.5; **(C)** PaO_2_/FiO_2_ (PF) >300 mmHg vs. 200 mmHg < PF ≤ 300 mmHg vs. 100 mmHg < PF ≤ 200 mmHg vs. PF ≤ 100 mmHg); **(D)** with or without mechanical ventilation support. LASSO, least absolute shrinkage and selection operator; INR, international normalized ratio.

Further, we evaluated the prognostic value of the use of vasopressor, INR > 1.5, the severity of impaired pulmonary function, and qSOFA score for the patients with Sepsis-3.0 who received CRRT. A significant difference in clinical outcomes was observed between with and without vasopressor support (Figure 4A, *p* < 0.001), INR > 1.5 compared with INR ≤ 1.5 (Figure 4B, *p* = 0.012), among different severity of impaired pulmonary function indicated with the value of PaO_2_/FiO_2_ (Figure 4C, *p* < 0.001), and with or without MV support (Figure 4D, *p* < 0.001).

## Discussion

Sepsis-induced aggressive hemodynamic alterations are one of the main causes for high mortality in patients with sepsis. CRRT, as a recommended management for hemodynamic stable, is paid more attention in recent years. In the present study, the retrospective study based on MIMIC-III v1.4 developed a *Cox-LASSO* model to show that use of vasopressor and INR ≥ 1.5 are found to be risk factors of hospital mortality in patients with sepsis who received CRRT. These findings may assist clinicians in tailoring precise management and therapy for these patients who underwent CRRT.

According to the international guideline for the management of sepsis in 2016 (24), CRRT is suggested to be used to facilitate the management of fluid balance in hemodynamically unstable septic patients. In the present study, the ratio of CRRT support in patients who met the criteria of Sepsis-3.0 was 2.6% (312/11791). There were about 5–6% of ICU patients with AKI who will receive RRT (25). This result is much lower than the ratio of CRRT application in patients with sepsis in adult ICU in China (16.3%) (26) and in pediatric ICU according to our previous study (10.7%) (27). As reported, the ratio of CRRT used in a patient with COVID-19-induced that AKI was from 3.9% (225/5700) to 8.7% (280/3235) in the USA (28). MIMIC-III v1.4 did not include detailed information about the indications for CRRT in each patient. According to the baseline characteristics of patients with sepsis who received CRRT, there were high ratio (70.7%, 128/181) of systolic BP <100 mmHg, high lactate levels (≥ 4 μmol/L) (46.4%, 84/181), high ratio of vasopressor support (39.8%, 72/181), and a high ratio of MV support (44.2%, 80/181). Miao et al. (29) reported the indications of CRRT in pediatric severe sepsis (http://links.lww.com/CCM/E733). Whether there are more detailed differences in CRRT application between pediatric and adult patients with sepsis who need further investigation in a well-designed study.

In adult ICU, hospital mortality and 28-day mortality ranged from 50.4 to 64.5% in patients with septic AKI under CRRT (30, 31). The recent insights and results from large randomized and non-randomized trials in the area of RRT applied for sepsis-induced AKI do not seem to improve survival or renal recovery (32). Our previous study indicated that CRRT decreases hospital mortality rate from 32.4% (44/136) to 21.3% (29/136) in pediatric patients with severe sepsis (29). Moreover, there is no evidence to indicate that high-volume hemofiltration (HVHF) is superior to standard-volume continuous veno-venous hemofiltration (CVVH) in the aspect of reducing 28-day mortality in pediatric patients with severe sepsis (24.7 vs. 33.8%, *p* = 0.216) (33). In our present study, the total hospital mortality was 17.1% (31/181). In the subgroup, the hospital mortality was 22.6% (19/84) in patients with lactate ≥ 4 μmol/L, 22.6% (29/128) with systolic BP <100 mmHg, 31.3% with MV support, and 36.1% (26/72) with vasopressor support. These findings give an overview of the clinical benefits of CRRT in adult sepsis.

Hypotension or the need for vasoactive drugs was associated with mortality (34). In our present study, the need for vasoactive drugs, but not hypotension (defined as systolic BP <100 mmHg), on ICU admission was entered the *LASSO* model for mortality in a patient with sepsis under CRRT. In addition, thrombocytopenia prior to the initiation of CRRT and severe thrombocytopenia prior to and following the initiation of CRRT were associated with increased ICU mortality (35). In the present study, though the ratio of patients with platelets <100 × 10^9^/L was higher in non-survivors than survivors, the ratio of patients with platelets <100 × 10^9^/L on admission was not an independent factor for mortality in patients under CRRT support. Moreover, AKI is a main indication for CRRT initiation, but the levels of serum creatinine were relatively lower in non-survivors than survivors, and there were no differences in the levels of BUN between the two groups. Consistently, there was a report that the severity of the AKI at the time of CRRT start did not have a significant relationship with the burned patient outcome with CRRT (36). Otherwise, in sepsis patients with AKI treated with CRRT, age, Acute Physiology and Chronic Health Evaluation (APACHE) II, SOAF, and grade IV of cardiac function were independent risk factors for death (37). In this study, qSOFA score was associated with mortality in patients treated with CRRT.

There are several limitations in this study. Firstly, we could not collect the detailed information about fluid overload in patients with sepsis. Secondly, the indications for CRRT were lacking in this study. Thirdly, as a database study, the interval time between sepsis occurrence and CRRT initiation was lacking. All these limitations could lead to bias for the present conclusions of this study, which needs further confirmation in a well-designed prospective study.

## Conclusions

In summary, we found that the use of vasopressor, INR ≥ 1.5, and qSOFA score are outcome of patients with sepsis who received CRRT based on MIMIC-III v1.4. After adjusted by qSOFA score, either lactate level or MV support is independently associated with the hospital mortality. These findings may assist clinicians in tailoring precise management of hemodynamics and coagulation disorders for these patients who underwent CRRT.

## Data Availability Statement

The datasets presented in this study can be found in online repositories. The names of the repository/repositories and accession number(s) can be found in the article/Supplementary Material.

## Ethics Statement

Ethical review and approval was not required for the study on human participants in accordance with the local legislation and institutional requirements. Written informed consent for participation was not required for this study in accordance with the national legislation and the institutional requirements.

## Author Contributions

CW and YZ conceptualized the research aims. CW planned the analyses, guided the literature review, and drafted the manuscript. JZ extracted the data from the MIMIC-III database. CW, JZ, and JW participated in processing the data and doing the statistical analysis. LZ and YZ provided comments and approved the final manuscript. All authors read and approved the final manuscript.

## Funding

This work was supported by the National Natural Science Foundation of China (82171729), the Natural Science Foundation of Shanghai (19ZR1442500), and National Key R&D Program of China (2020YFC2005802 and 2020YFC2005800). CW was funded by the Shanghai Municipal Education Commission-Gaofeng Clinical Medicine Grant (20171928) and the talent program from Shanghai Jiao Tong University School of Medicine (17XJ11018).

## Conflict of Interest

The authors declare that the research was conducted in the absence of any commercial or financial relationships that could be construed as a potential conflict of interest.

## Publisher's Note

All claims expressed in this article are solely those of the authors and do not necessarily represent those of their affiliated organizations, or those of the publisher, the editors and the reviewers. Any product that may be evaluated in this article, or claim that may be made by its manufacturer, is not guaranteed or endorsed by the publisher.

## Abbreviations

BP: blood pressure
BUN: blood urea nitrogen
*CI*: confidence intervals
CRRT: continuous renal replacement therapy
*HR*: hazard ratio
ICD: International Classification of Diseases
ICU: intensive care unit
INR: international normalized ratio
LODS: Logistic Organ Dysfunction System
MIMIC: Medical Information Mart for Intensive Care
PaO_2_/FiO_2_: the ratio of the partial pressure of oxygen in arterial blood (PaO_2_) to the inspired oxygen fraction (FiO_2_)
qSOFA: quick sequential organ failure assessment
SIRS: systemic inflammatory response syndrome
SOFA: sequential organ failure assessment
SpO_2_: oxyhemoglobin saturation
SSC: surviving sepsis campaign
WBC: white blood cells.
